# Pediatric Dentists’ Practice Patterns in the Management of Permanent Teeth Needing Endodontic Treatment

**DOI:** 10.3390/dj13050191

**Published:** 2025-04-26

**Authors:** Nuha Ashraf, Linda Sangalli, Jackson Seagroves, Caroline M. Sawicki

**Affiliations:** 1 Department of Pediatric Dentistry and Dental Public Health, Adams School of Dentistry, Chapel Hill, NC 27517, USA; nashraf@unc.edu; 2 College of Dental Medicine–Illinois, Midwestern University, Downers Grove, IL 60515, USA; lsanga@midwestern.edu; 3 Department of Periodontology, Endodontics, and Dental Hygiene, Adams School of Dentistry, Chapel Hill, NC 27517, USA; jseagrov@live.unc.edu

**Keywords:** dental education, endodontics, pediatric dentistry

## Abstract

**Background/Objectives**: The objective of this study was to evaluate practice patterns among pediatric dentists for the management of permanent teeth needing endodontic treatment. **Methods**: An electronic nationwide survey was sent to all members of the American Academy of Pediatric Dentistry (AAPD). The survey assessed provider training on and confidence (0–100, with 100 = “most confident”) in treating pediatric patients needing endodontic treatment on permanent teeth, referral patterns, and preferred educational resources. A logistic regression identified significant predictors of confidence levels. **Results:** The final sample included 259 respondents, with 71% having over 10 years of experience in practice. A total of 47% of respondents reported performing endodontic treatments on permanent teeth in pediatric patients, with direct pulp capping (70%) and partial or full pulpotomy (62%) being the most common procedures. Although the respondents moderately agreed (53 ± 32) that they received sufficient training during their residency to perform endodontic treatment on permanent teeth, their reported comfort levels with performing these procedures were low (0.001 ± 33). The adequacy of the training received during their residency was identified as a significant predictor of a higher level of confidence (*p* < 0.001, 95% CI 0.437, 0.667). The respondents highlighted additional continuing education courses and training, dedicated lectures at the AAPD Annual Session, and annual joint symposia or meetings between the AAPD and the American Association of Endodontists as the most valuable educational resources for the endodontic management of permanent teeth in pediatric patients. **Conclusions**: The findings suggest that enhancing residency training and expanding access to targeted continuing education opportunities are critical for improving pediatric dentists’ confidence and competence in the endodontic management of permanent teeth in pediatric patients.

## 1. Introduction

Endodontic procedures are both prevalent and clinically significant in pediatric dentistry, reflecting the high incidence of caries- and trauma-related pulpal pathology in both primary and permanent dentitions [1]. In the primary dentition, the prevalence of pulpitis has been estimated at approximately 1.55 cases per child, with nearly 50% of children requiring endodontic therapy having undergone at least two clinical endodontic procedures [2]. In comparison, endodontic treatment needs affect up to 9.6% of pediatric patients overall, with permanent teeth accounting for nearly 70% of these cases [3,4]. Caries and traumatic dental injuries are the primary reasons for endodontic treatment in permanent teeth, with prevalences of approximately 64% and 33%, respectively [3]. Endodontic treatment in pediatric patients presents unique challenges due to the anatomical and physiological characteristics of developing teeth, the complexities of behavior management, and the technique-sensitive nature of the procedures, which require specialized materials not commonly used in everyday practice. Importantly, endodontic treatment in primary teeth is typically limited to less complex procedures, such as pulpotomy or pulpectomy, often with a focus on maintaining function until exfoliation [1]. In contrast, permanent teeth require more intricate and definitive therapies, such as apexogenesis, apexification, or root canal therapy, to preserve long-term tooth viability [1]. Treating permanent teeth in pediatric patients presents additional difficulties, including anesthetic management, the potential need for multiple visits to complete treatment, and the prolonged procedure duration [3]. In addition, successful management requires an integration of clinical expertise and tailored treatment strategies to address patient anxiety and non-compliance. Pediatric dentists play a critical role in endodontic management, balancing pulp preservation with long-term restorative outcomes. However, clinical practice patterns for the endodontic management of permanent teeth vary among pediatric dentists.

A notable gap exists between the American Academy of Pediatric Dentistry (AAPD) clinical guidelines and the Commission on Dental Accreditation (CODA) standards in preparing pediatric dentists for managing endodontic procedures in their patients. While AAPD guidelines provide clinical recommendations for pulp therapy in immature permanent teeth, from protective liners to regenerative endodontics [1], CODA standards fall short in fully integrating these competencies into residency training [5]. This discrepancy raises concerns about whether pediatric dental residents receive sufficient clinical and didactic instruction to confidently manage dental pain and both vital and non-vital pulp conditions in permanent teeth upon the program’s completion. This, in turn, directly impacts their confidence, which is a precursor to implementation. Self-perceived confidence refers to “confidence in one’s abilities, qualities and judgements” [6]. It is positively associated with educational development and academic performance [7] and results from educational experience [8]. Hence, confidence in performing endodontic procedures on permanent teeth may be influenced by factors such didactic instruction and clinical experience during residency training, as well as access to continuing education.

The AAPD and American Association of Endodontists (AAE) emphasize the need for standardized, effective pulp therapy practices across both specialties, ensuring that pediatric dentists feel competent to manage pulpal diseases in permanent teeth [9]. Alignment in guidelines, both in language and content, is necessary to ensure consistency in performing pulp therapies and to optimize patient outcomes. However, a significant educational gap may undermine these efforts, leading to reduced confidence among graduating pediatric dentistry residents in performing endodontic procedures on permanent teeth. This lack of confidence often leads to increased referrals to endodontists, which can delay treatment and elevate healthcare costs, disproportionately affecting children from lower socioeconomic backgrounds or those on public insurance with limited access to specialized care [10]. Furthermore, it is not clear to what extent endodontists are trained to adequately manage pediatric patients, since pediatric dental care falls primarily within the scope of pediatric dentistry. In fact, studies have shown that 13% of endodontists do not even provide treatment for pediatric patients [11]. These disparities further highlight the need for pediatric dentistry residency curricula revisions that better align with AAPD guidelines, ensuring all pediatric dentists are adequately trained to manage dental pain and both vital and non-vital pulp conditions in permanent teeth effectively.

However, addressing this educational gap requires more than just recognition; it necessitates both policy reforms and advancements in dental education. Pediatric dentists frequently treat patients covered by public payer dental insurance (e.g., Medicaid or Children’s Health Insurance Program), who often encounter significant barriers to accessing endodontic care [12]. Notably, recent studies show that only 17% of endodontists participate with public-payer dental insurance [10]. Given the limited access to endodontists, it is crucial to equip pediatric dentists with the competencies needed to manage endodontic cases effectively. Enhancing pediatric dentists’ proficiency in endodontic procedures can minimize referrals, streamline treatment, and expand access to care for underserved pediatric populations.

Expanding access to pediatric endodontic care depends on strengthening pediatric dentistry residency programs to offer more extensive endodontic training. This study aimed to evaluate practice patterns in the endodontic management of permanent teeth among pediatric dentists and residents. We hypothesized that most pediatric dentists would lack confidence in performing endodontic treatment on permanent teeth, likely due to gaps in their residency training and limited continuing education opportunities.

## 2. Materials and Methods

### 2.1. Study Design

The cross-sectional survey was reviewed by the University of North Carolina Institutional Review Board (IRB) and deemed to be exempt (IRB #24-2649, 3 November 2024). An anonymous online questionnaire was distributed via Qualtrics to all members of the AAPD, with email reminders at two-week intervals for a total of four requests over a two-month study period (from November to December 2024). Eligible participants needed to be in the process of completing or have had already completed specialized training in pediatric dentistry. Electronic informed consent was obtained from respondents prior to participation. In accordance with local IRB regulations, participants were not obligated to respond to all survey items.

### 2.2. Survey Assessment Tool

The anonymous surveys (Supplemental File S1) were co-developed by a pediatric dentist (C.M.S.) and endodontist (J.S.) who are specialized in pediatric dentistry and endodontics, respectively. Survey content was also reviewed by external content experts to ensure relevance and appropriateness of questions, as well as readability and accessibility. The final survey consisted of 16 items across 4 sections (Table 1). Responses to all sections were optional. Section 1 examined respondents’ clinical encounters, practice approaches (direct pulp cap, partial or full pulpotomy, root canal therapy, apexogenesis or apexification, revascularization, or pulpal regeneration) in managing permanent teeth requiring endodontic treatment, and referrals (frequency, type of provider, and reasons for referral). Section 2 evaluated respondents’ management of traumatic dental injuries in permanent teeth (frequency, age group, and preferred management choice). Section 3 gathered information regarding respondents’ interest in obtaining educational resources pertaining to endodontic management of permanent teeth in pediatric patients. Finally, Section 4 assessed respondents’ levels of comfort with and perceived training on (on a 0–100 scale, with 100 = “Strongly agree”) performing endodontic treatment on permanent teeth and managing traumatic dental injuries. Opportunities to elaborate on close-ended responses were provided throughout via optional open text boxes, and the last item of the survey was an open text box with an invitation to “share any other thoughts or feedback related to the management of pediatric endodontic cases involving permanent dentition”. The survey also assessed sociodemographic (age, sex, race/ethnicity, practice location, and additional training) and professional characteristics (years of practice, number of working days per week, and type of clinical setting).

### 2.3. Statistical Analysis

Descriptive statistics (means and standard deviation for continuous data and frequency and percentage for categorical data) were used to describe the sample.

Chi-square tests were used to investigate whether the likelihood of performing endodontic treatments for permanent teeth in pediatric patients was correlated with the number of years after the providers’ graduation. Independent *t*-tests were used to evaluate whether or not performing such endodontic treatments was influenced by self-perceived confidence levels in managing these treatments. Effect sizes were computed with Cohen’s *d* [13]. Confidence levels were compared among respondents with different percentages of referral to outside providers using ANOVA with Bonferroni’s post hoc test. Multiple regression analysis was used to test whether confidence levels in performing endodontic treatment on permanent teeth were influenced by the number of years of practice and training received during residency.

A thematic analysis was performed to categorize the reasons for outside referral of endodontic management of permanent teeth into most common patterns (themes) with frequency counts.

## 3. Results

### 3.1. Sample Description

A total of 5800 AAPD members were invited to participate in the study. Responses were collected from 268 participants; however, seven responses were left empty and two participants indicated they had not completed or were not attending specialized training in pediatric dentistry in a CODA-approved program. Thus, the final analysis included N = 259 respondents (a 4.5% response rate).

Table 2 shows the demographic data, professional attributes, and educational background of the 259 respondents. The participants were nearly evenly distributed between females and males (46.3% vs. 51.0%, respectively). Approximately half of them were between 36 and 55 years old. Most respondents identified their ethnicity as non-Hispanic or Latinx (85.7%) and their race as White (73.4%). As high as 71.0% of the respondents had over 10 years of practice.

### 3.2. Practice Patterns Regarding Endodontic Management of Permanent Teeth

**Figure 1:**
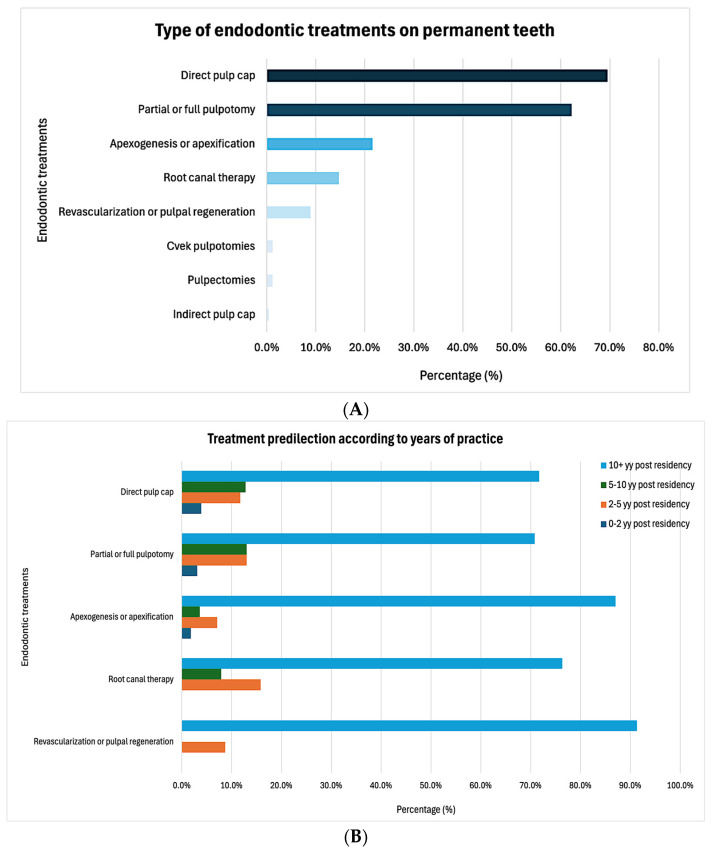

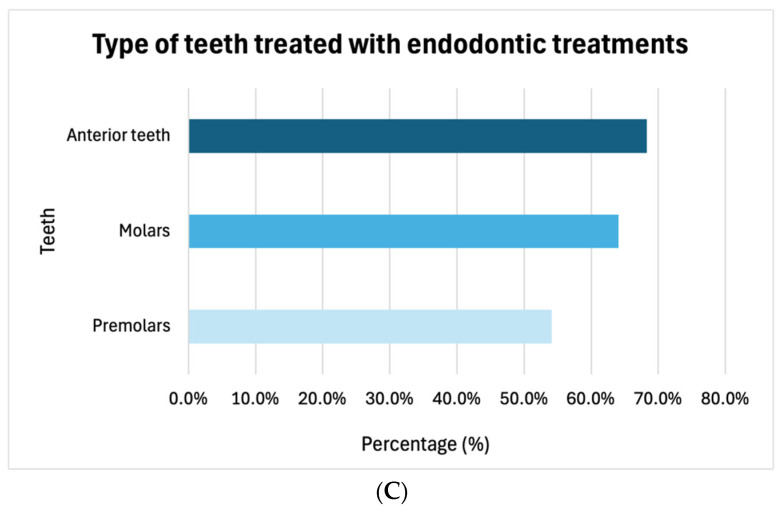
Practice patterns regarding endodontic management: (**A**) type of endodontic treatments performed, (**B**) influence of years of experience, and (**C**) type of teeth.

**Figure 2:**
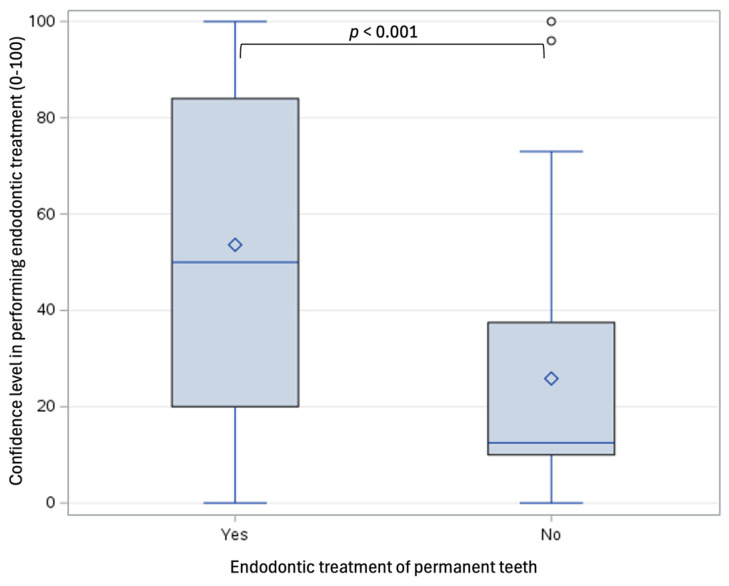
Influence of self-perceived confidence levels on the likelihood of performing endodontic treatment of permanent teeth.

The majority of the respondents (76.8%) reported performing endodontic treatments on permanent teeth in pediatric patients, most commonly consisting of direct pulp capping (69.5%) and partial or full pulpotomy (62.2%, Figure 1A). Most of these treatments were performed by providers with over 10 years of experience (Figure 1B). These treatments were most frequently performed on anterior teeth (68.3%), followed by molars (64.1%, Figure 1C). There was a moderate level of agreement among the respondents (53.4 ± 31.8) regarding having received adequate training during their pediatric dentistry residency on performing endodontic treatment on permanent teeth in pediatric patients. However, their comfort levels with performing such procedures were even lower (47.3 ± 33.1). Pediatric dentists who did not perform endodontic treatments on permanent teeth in pediatric patients reported significantly lower comfort levels compared to those who performed these treatments (25.8 ± 23.4 vs. 53.6 ± 32.5, *p* < 0.001, Cohen’s d = 0.90, Figure 2). There was no correlation between the likelihood of performing endodontic treatments on permanent teeth in pediatric patients and the number of years the providers had been practicing (X^2^(3) = 0.796, *p* = 0.850).

### 3.3. Referral Practices for Endodontic Treatment of Permanent Teeth

**Figure 3:**
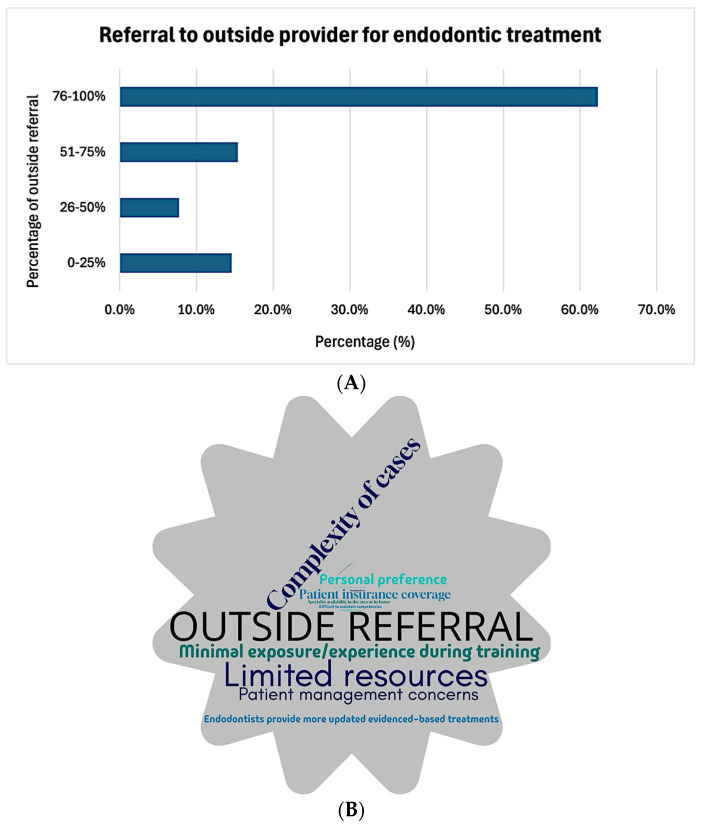
Practice patterns regarding referral of endodontic management of permanent teeth: (**A**) frequency of referral to outside providers and (**B**) word cloud depicting the reasons for referral to outside providers. The size of each word reflects its frequency, with larger statements being more often reported by the respondents. This thematic analysis derives from the responses to the item “What are the main reasons for referring pediatric endodontic cases involving permanent dentition out of your practice?”.

**Figure 4:**
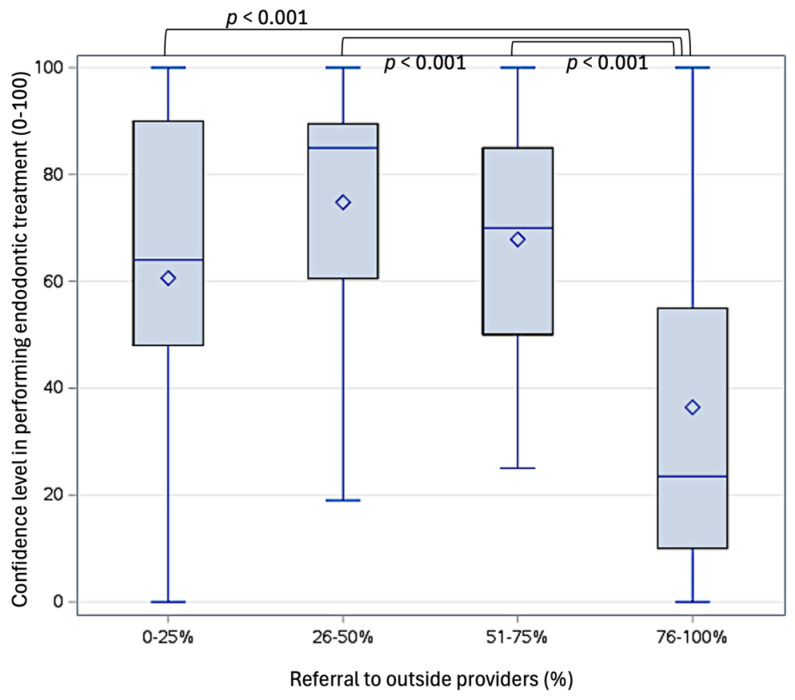
Influence of self-perceived confidence levels on percentage of referrals of endodontic management of permanent teeth to outside providers.

Almost two-third of the providers (62.3%) reported referring more than 75% of their pediatric patients to outside specialists for endodontic treatment of permanent teeth (Figure 3A), most commonly to endodontists (21.2%). As illustrated in Figure 3B, the main reasons for referrals were (in descending order) the complexity of these cases (65.6%), having had minimal exposure to or experience in endodontics during their pediatric dentistry residency (36.7%), limited resources (31.3%), patient management concerns (8.5%), personal preference (7.2%), and patient insurance coverage (6.2%). All other reasons were cited by fewer than 5% of the respondents. There was no significant correlation between the number of years of practice and the frequency of referrals (X^2^(9) = 10.322, *p* = 0.325). However, referral patterns varied significantly based on self-perceived comfort levels in performing endodontic treatments on permanent teeth in pediatric patients (F(3, 226) = 18.810, *p* < 0.001). The providers referring 76–100% of their patients expressed the lowest confidence levels (36.4 ± 30.5) compared to those referring 0–25% (60.6 ± 34.8, *p* < 0.001), 26–50% (74.8 ± 24.8, *p* < 0.001), and 51–75% of their patients (67.9 ± 22.6, *p* < 0.001, Figure 4).

Years of practice and self-perceived adequacy of training received during the residency program accounted for 28.6% of the variance in confidence levels. While years of practice did not have a statistically significant effect on self-reported confidence levels, the adequacy of training received during residency emerged as significant predictor of increased confidence (*p* < 0.001, 95% CI 0.437, 0.667). Specifically, each additional point indicating more sufficient training in performing endodontic treatment was associated with a 0.55-unit increase in confidence levels.

### 3.4. Practice Patterns Regarding Traumatic Dental Injuries

**Figure 5:**
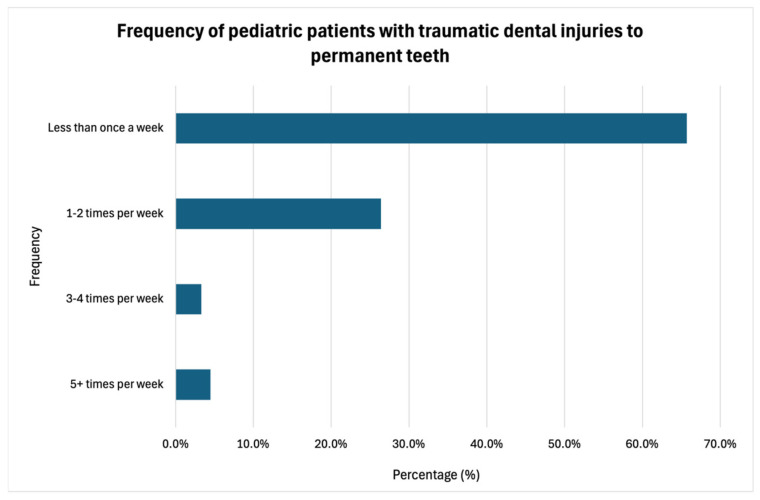
Practice patterns regarding traumatic dental injuries.

Most of the respondents (61.4%) reported encountering traumatic dental injuries to permanent teeth in pediatric patients less than once per week, while 24.7% indicated seeing such cases one to two times per week (Figure 5). Among these cases, 82.2% required endodontic treatment in less than 25% of incidents. The most commonly affected age group was 9–12 years, as reported by 74.4% of the respondents. When managing permanent dentition trauma requiring endodontic treatment, 84.7% of the respondents indicated they routinely perform an initial consultation and evaluation, while 12.4% did so occasionally.

The respondents reported a relatively high level of confidence (83.9 ± 20.0) in managing traumatic dental injuries involving permanent teeth in pediatric patients.

### 3.5. Educational Resources

Continuing education courses (73.7%) and scientific conferences (36.7%) were identified as the most common sources of additional training relevant to managing endodontic treatment on permanent teeth (Table 3). The respondents demonstrated a moderate interest (62.3 ± 33.9) in learning more about managing pediatric endodontic cases involving permanent dentition. The most helpful educational resources were identified as (in descending order) continuing education courses and training (62.5%), more dedicated lectures at the AAPD Annual Session (40.9%), annual joint symposia or meetings between AAPD and the American Association of Endodontics (AAE, 37.1%), and research on the prevalence of these cases (15.8%). Conversely, 5.6% of the respondents expressed no interest in further education, preferring to refer patients to outside specialists (Table 2). The pediatric dentists who performed endodontic treatments on permanent teeth were significantly more interested in enhancing their knowledge on managing such cases compared to those who did not perform these treatments (65.7 ± 32.4 vs. 50.8 ± 36.6, *p* = 0.005, Cohen’s d = 0.45).

## 4. Discussion

The findings of this study highlight a critical issue in pediatric dentistry regarding the endodontic management of permanent teeth in pediatric patients. The survey responses from 259 pediatric dentists and residents reveal substantial variability in confidence and practice patterns, emphasizing a gap between current training and clinical demands. Notably, confidence levels were strongly correlated with the perceived adequacy of residency training, which emerged as a significant predictor of higher confidence (*p* < 0.001). These findings highlight the need for enhanced residency training and expanded continuing education opportunities to better prepare pediatric dentists for managing endodontic cases in permanent teeth.

A significant portion of the pediatric dentist respondents (62%) indicated that they refer most endodontic cases, with procedural complexity and insufficient residency training being cited as key factors. This aligns with prior reports that pediatric dentists are less likely than endodontists to select regenerative procedures for necrotic immature permanent teeth, a difference attributed in part to variations in residency training and exposure to continuing education. Our findings highlight a critical gap in the current training and preparation of pediatric dentists, particularly when it comes to managing complex endodontic procedures in permanent teeth. The fact that a majority of respondents choose to refer cases suggests a lack of confidence or perceived competence in performing these procedures, which can ultimately contribute to delayed treatment and increased healthcare costs. Limited confidence in handling endodontic procedures was evident in the current study, in which the respondents who referred 76–100% of their patients reported the lowest confidence levels compared to those who referred fewer cases (0–75%). Furthermore, the lack of sufficient residency training, as indicated by 36.7% of the respondents, points to an educational shortfall in preparing pediatric dentists for the specific clinical demands of endodontic management in permanent teeth.

Current findings indicate that the adequacy of training received during residency is a significant predictor of increased confidence in managing pediatric endodontic cases involving permanent teeth. This gap in training could be attributed to limited exposure to endodontic procedures during residency or a focus on other aspects of pediatric dentistry that may not prioritize endodontic treatment in permanent teeth. These findings highlight the necessity for residency programs to expand their curriculum to include more comprehensive training in endodontics, ensuring that pediatric dentists are better equipped to handle such cases in clinical practice. Additionally, increasing interdisciplinary interactions during residency training, as required by the CODA for endodontics [14], could help bridge the gap in endodontic clinical proficiency among pediatric dentists and improve pediatric patient management skills among endodontists. Previous studies have demonstrated inconsistent clinical philosophies between pediatric dentists and endodontists regarding vital pulp therapy, particularly in immature permanent teeth, highlighting the importance of harmonizing approaches across both specialties to ensure consistent treatment outcomes [9]. Promoting collaboration and integrated training, especially at institutions with both endodontics and pediatric dentistry postgraduate programs, could create a mutually beneficial learning environment that strengthens clinical competency in both specialties. In addition to training deficiencies, the structural barriers faced by pediatric dentists also influence referral practices and highlight a broader systemic issue within the pediatric dentistry education system. Without adequate training in endodontics, particularly for cases involving permanent teeth, pediatric dentists may become overly reliant on specialists, further straining the availability and accessibility of care. Additionally, pediatric dentists face significant challenges in referring pediatric patients to endodontists, primarily due to insurance barriers and a shortage of providers accepting Medicaid or other public-payer dental insurance plans [10]. Previous studies reveal substantial disparities in the provision and outcomes of dental care based on insurance type [15]. While children with public-payer dental insurance are more likely to receive endodontic treatments, such as root canal therapy, they experience poorer outcomes compared to their privately insured counterparts [16]. Children covered by public-payer dental insurance are particularly disadvantaged, receiving less frequent care from dental specialists and often in less-equipped facilities, which leads to higher rates of treatment failure and adverse outcomes [16]. Despite having seemingly equal access to care, there are significant disparities in the quality and frequency of endodontic treatments, with significant implications for the long-term oral health of children with public insurance [17]. The reliance on referrals thus exacerbates treatment inefficiencies, resulting in longer wait times and higher costs. These barriers are especially harmful for children in underserved areas who have limited access to specialized care. While the previous literature has documented the disproportionate burden faced by publicly insured pediatric patients in accessing specialty dental care [15], our study extends these findings by quantifying how provider confidence, shaped by training, directly contributes to referral patterns. This reinforces prior reports of outcome disparities tied to insurance status [15] and suggests a structural cycle in which both educational and systemic barriers compound access limitations. To address the educational gaps identified in this study, one possible solution may be to explore the integration of virtual learning experiences into pediatric endodontic training. Virtual reality and simulation-based learning platforms offer innovative solutions that could provide pediatric dentists with immersive, hands-on practice environments, eliminating the risks associated with real-life procedures [18,19]. These technologies can allow pediatric dental residents to practice endodontic treatments on permanent teeth, ranging from routine pulpotomies to more complex cases involving severe dental trauma, within a controlled setting. Such virtual training could enhance skill development, build confidence, and ensure the consistent application of theoretical knowledge [18,19,20]. Notably, Slaczka et al. demonstrated that learners trained using Simodont, a virtual reality dental trainer, showed skill progression comparable to those trained through traditional methods [21]. This finding highlights the efficacy of virtual reality as a training tool, particularly for intricate procedures like endodontic access cavity preparation. Given its potential, virtual-reality-based training offers a promising strategy for enhancing the competency of pediatric dentists in managing endodontic cases effectively. Building on the potential of virtual reality as an immersive and hands-on training tool, the success of multimodal educational frameworks, such as the Dental Sleep Medicine Mini-Residency at Tufts University, further highlights the value of integrating diverse learning methods [22]. Combining theoretical learning with clinical practice and self-study could not only improve pediatric dentists’ procedural competence but also greatly increase their confidence in managing complex endodontic cases. Such integrated approaches have demonstrated substantial improvements in both knowledge and professional behavior [22], proving to be an effective model for addressing the gaps in pediatric endodontic training identified in this study.

Due to the significant gaps in confidence and practice patterns in the endodontic management of permanent teeth highlighted by this study, 73.7% of the respondents indicated a strong interest in pursuing additional training opportunities. This need is especially pressing, as only 47% of the pediatric dentists reported confidence in performing vital pulp therapies in permanent teeth, despite 76.8% actively performing these procedures. Additionally, over a third of the respondents expressed interest in attending joint symposia between the AAPD and AAE, highlighting the benefit of such forums in enhancing pediatric endodontic knowledge. These symposia should focus on reinforcing evidence-based practices in pulpal therapy, aligning guidelines across both specialties, and addressing the specific challenges pediatric dentists face in managing endodontic cases. Increasing access to targeted educational initiatives, such as joint symposia, can improve confidence, minimize unnecessary referrals, and ensure more effective care for pediatric patients, particularly those with limited access to specialized endodontic care.

Additionally, the discrepancies between the standards set by the CODA and the guidelines released by the AAPD have significant implications for the confidence and clinical effectiveness of pediatric dentists. For example, AAPD guidelines provide a brief overview of how to perform vital pulp therapies (e.g., protective liners, apexogenesis, direct pulp capping, and complete pulpotomy) and non-vital pulp treatments (e.g., root canal treatment, apexification, and regenerative endodontics) for immature permanent teeth, suggesting that pediatric dentists already have the foundational clinical knowledge and competency to perform these procedures. CODA standards for pediatric dentistry state that residents must achieve clinical competency in vital and non-vital pulp therapy in immature permanent teeth, yet over a third of respondents in the current study indicated that having minimal exposure to or experience in endodontics during their residency was a significant reason for referring out these cases. Addressing these educational and systemic gaps is essential to align CODA standards with AAPD guidelines, ensuring pediatric dentists are well equipped to manage endodontic cases in permanent teeth. By incorporating multimodal educational frameworks during residency training, pediatric dentists can gain the necessary skills and confidence to effectively manage complex endodontic cases, from pulpotomies in permanent teeth to more complex trauma-related procedures, ultimately addressing the significant gaps in training and improving overall treatment outcomes for pediatric patients.

Several limitations must be considered when interpreting the study’s findings. The main limitation was the low response rate, despite the authors’ repeated efforts including reminder emails and information regarding the surveys’ short completion time [23]. With only 259 respondents, the findings may have limited generalizability to the broader pediatric dental community. The low participation rate also introduces potential selection bias, as those who responded may not accurately represent pediatric dentists with varying levels of exposure to or confidence in endodontic procedures. Moreover, all information was self-reported; thus, providers’ behaviors were not evaluated directly. Last, as the study only focused on pediatric dental providers who were members of the AAPD, the findings may not be applicable to pediatric dentists who are not involved in the AAPD.

Future research should explore objective assessments of clinical competence and track clinical practice patterns associated with pediatric dentists who receive enhanced endodontic training during their residency or through continuing education. Longitudinal studies are also needed to evaluate the sustained impact of training interventions, such as simulation-based learning, joint specialty symposia, or integrated rotations with endodontics, on provider confidence and referral patterns. Additionally, qualitative investigations may provide valuable insights into the perceived barriers to providing endodontic treatment for permanent teeth, including time constraints, institutional support, and clinical mentorship. Importantly, future research should also examine patient-level outcomes and healthcare utilization in relation to training models, with particular attention to vulnerable populations such as children with public insurance. These efforts are essential for developing scalable educational reforms that not only enhance provider competence but also reduce access disparities for pediatric patients requiring endodontic treatment.

## 5. Conclusions

The current study reveals significant gaps in pediatric dentists’ training and confidence in managing endodontic procedures in permanent teeth, with inadequate residency training emerging as a key factor influencing high referral rates. These trends contribute to delayed treatment and reduced access to care, particularly for publicly insured children. Enhancing residency curricula, increasing interdisciplinary training, and expanding access to continuing education are essential to improve clinical competence. Integrating innovative approaches such as simulation-based learning and joint specialty symposia may further bolster provider preparedness and confidence. Addressing the educational deficiencies identified in this study is critical for enhancing pediatric dentists’ ability to manage complex cases involving permanent teeth and for promoting equitable access to care for underserved populations.

## Figures and Tables

**Table 1:** Survey Questionnaire.

Domain	Item Question	Answer Options
Section 1		
Clinical encounters	Do you perform any endodontic treatments for permanent teeth in pediatric patients at your practice?	☐ Yes ☐ No
	Of the endodontic treatments you perform at your practice, which teeth do you treat?	☐ Anterior teeth ☐ Premolars ☐ Molars
Practice approaches in managing permanent teeth requiring endodontic treatment	What type of endodontic treatments on permanent teeth do you perform at your practice?	☐ Direct pulp cap ☐ Partial or full pulpotomy ☐ Root canal therapy ☐ Apexogenesis or apexification ☐ Revascularization or pulpal regeneration ☐ Other (please describe)
Referral	For your pediatric patients requiring endodontic treatment on permanent teeth, who do you usually refer them to?	☐ Endodontist ☐ General dentist ☐ Oral surgeon ☐ Another pediatric dentist ☐ Other (please describe)
	On average, what percentage of pediatric patients requiring endodontic treatment on permanent teeth do you refer to an outside provider?	☐ 0–25% ☐ 26–50% ☐ 51–75% ☐ 76–100%
	What are the main reasons for referring pediatric endodontic cases involving permanent dentition out of your practice?	☐ Complexity of the case ☐ Limited resources ☐ Patient management concerns ☐ Patient insurance coverage ☐ Minimal exposure/experience in endodontics during pediatric dentistry residency ☐ Other (please describe)
Section 2		
Management of traumatic dental injuries in permanent teeth	How often do you encounter pediatric patients with traumatic dental injuries to permanent teeth in your practice?	☐ Less than once a week ☐ 1–2 times per week ☐ 3–4 times per week ☐ 5+ times per week
	What percentage of trauma cases that you encounter involve the need for endodontic treatment on permanent teeth?	☐ 0–10% ☐ 11–25% ☐ 26–50% ☐ 51–75% ☐ 76–100%
	In your experience, what age group most commonly presents with traumatic injuries to permanent teeth requiring endodontic treatment?	☐ 6–8 years ☐ 9–12 years ☐ 13–16 years ☐ 17 years or older
	When a patient presents with a trauma in the permanent dentition involving need for endodontic treatment, do you perform the initial consultation and evaluation or do you immediately refer them to a specialist?	☐ I perform the initial consultation and evaluation ☐ I sometimes perform the initial consultation and evaluation, depending on the complexity of the case ☐ I immediately refer out to a specialist without an initial consultation ☐ Other (please describe)
Section 3		
Interest in educational resources	Which of these educational resources would be most helpful for you as a pediatric dentist to best manage pediatric endodontic cases involving permanent dentition?	☐ More studies to determine the prevalence of permanent teeth needing endodontic treatment in pediatric patients ☐ Continuing education courses and training focused on permanent teeth needing endodontic treatment in pediatric patients ☐ More lectures at the AAPD Annual Session dedicated to endodontic management of permanent teeth ☐ Annual joint symposia/meeting of AAPD and American Association of Endodontics (AAE)
	I would be interested in learning more about how to treat pediatric endodontic cases involving permanent dentition	Please state your agreement level from 0 = “Strongly disagree” to 100 = “Strongly agree”
Section 4		
Perceived training	Besides your pediatric dentistry residency, what additional training have you received that is relevant to managing permanent teeth that need endodontic treatment in pediatric patients?	☐ Additional postgraduate specialty training ☐ Continuing education courses ☐ Simulation training ☐ Attendance at national and/or international conferences ☐ Other (please describe)
Level of comfort in endodontic treatment on permanent teeth	I received sufficient training during my pediatric dentistry residency on performing endodontic treatment for permanent teeth in pediatric patients	Please state your agreement level from 0 = “Strongly disagree” to 100 = “Strongly agree”
	I feel comfortable performing endodontic treatment on permanent teeth in pediatric patients	
Level of comfort in endodontic treatment for traumatic dental injuries	I feel comfortable managing traumatic dental injuries involving permanent teeth in pediatric patients	Please state your agreement level from 0 = “Strongly disagree” to 100 = “Strongly agree”

**Table 2:** Demographics, job characteristics, and education of the total sample.

Variable	Response Options	Total Sample (N = 259)
Demographics		
Sex (N, %)	Female	120 (46.3)
	Male	132 (51.0)
	Non-binary/third gender	1 (0.4)
	Prefer not to answer	2 (0.8)
	Missing	4 (1.5)
Age group (N, %)	Between 25 and 35 years	32 (12.4)
	Between 36 and 45 years	62 (23.9)
	Between 46 and 55 years	68 (26.3)
	Between 56 and 65 years	50 (19.3)
	Over 66 years	42 (16.2)
	Prefer not to answer	1 (0.4)
	Missing	4 (1.5)
Ethnic background (N, %)	Hispanic or Latinx	15 (5.8)
	Not Hispanic or Latinx	222 (85.7)
	Unsure	4 (1.5)
	Prefer not to answer	14 (5.4)
	Missing	4 (1.5)
Racial background (N, %)	White	190 (73.4)
	Black or African American	11 (4.2)
	American Indian or Alaska Native	2 (0.8)
	Asian	31 (12.0)
	Native Hawaiian or Other Pacific Islander	1 (0.4)
	Other	11 (4.2)
	Unsure	2 (0.8)
	Prefer not to answer	7 (2.7)
	Missing	4 (1.5)
Job Characteristics		
Years of practice (N, %)	0–2 years post residency	8 (3.1)
	2–5 years post residency	30 (11.6)
	5–10 years post residency	33 (12.7)
	10+ years post residency	184 (71.0)
	Missing	4 (1.5)
Days per week of practice (N, %)	None	1 (0.4)
	Between 1 and 3 days/week	49 (19.2)
	Between 4 and 5 days/week	204 (78.8)
	6 or more days/week	1 (0.4)
	Missing	4 (1.5)
Type of clinical setting (N, %)	Private practice	201 (77.6)
	Academic institution	21 (8.1)
	Hospital	17 (6.6)
	Community clinic or federally qualified health center	9 (3.5)
	Other	7 (2.7)
	Missing	4 (1.5)
Job location (N, %)	Northeast	69 (26.6)
	Midwest	52 (20.1)
	West	48 (18.5)
	Southeast	46 (17.8)
	Southwest	40 (15.4)
	Missing	4 (1.5)
Additional Educational Qualification		
Additional qualification (N, %) ^1^	General Practice Residency	39 (15.1)
	Orthodontics	21 (8.1)
	Advanced Education in General Dentistry	12 (4.6)
	Dental Public Health	11 (4.2)
	Dental Anesthesiology	4 (1.5)
	Endodontics	4 (1.5)
	Oral Medicine	1 (0.4)
	Oral and Maxillofacial Surgery	1 (0.4)
	Prosthodontics	1 (0.4)
	Other	10 (3.9)
	None	168 (64.9)

^1^ Note: Respondents could choose more than one option.

**Table 3:** Additional training and educational resources identified by the respondents to best manage pediatric endodontic cases involving permanent dentition.

Variable	Response Options	Total Sample (N = 259)
Additional training (N, %) ^1^	Continuing education courses	191 (73.7%)
	National and/or international conferences	95 (36.7%)
	Additional postgraduate specialty training	30 (11.6%)
	Simulation training	13 (5.0%)
	Collaboration with endodontists and clinical experience	8 (3.2%)
	Clerkship in endodontics	1 (0.4%)
	Research in endodontics	1 (0.4%)
	Other	1 (0.4%)
Educational resource (N, %) ^1^	Continuing education courses	162 (62.5%)
	Dedicated lectures at AAPD Annual Session	106 (40.9%)
	Annual joint symposia/meetings between AAPD and AAE	96 (37.1%)
	Studies on prevalence of permanent teeth needing endodontic treatment in pediatric patients	41 (15.8%)
	Dedicated training during residency	2 (0.8%)
	No interest	15 (5.6%)

^1^ Note: Respondents could choose more than one option. AAE: American Association of Endodontists. AAPD: American Academy of Pediatric Dentistry.

## Data Availability

The original contributions presented in the study are included in the article; further inquiries can be directed to the corresponding author.

## Supplementary Materials

The following supporting information can be downloaded at: https://www.mdpi.com/article/10.3390/dj13050191/s1, Supplementary File S1: Survey Questionnaire.

## Abbreviations

The following abbreviations are used in this manuscript:

AAE	American Association of Endodontists
AAPD	American Academy of Pediatric Dentistry
CODA	Commission on Dental Accreditation
