# Supplementary material for: Pediatric Dentists’ Practice Patterns in the Management of Permanent Teeth Needing Endodontic Treatment
Source: Dent J (Basel). 2025 Apr 26;13(5):191. doi: 10.3390/dj13050191 (PMC12109604; doi:10.3390/dj13050191)
Supplement: Supplementary file 1 [file dentistry-13-00191-s001.zip › dentistry-3568845-SI.pdf]

UNC SCHOOL OF DENTISTRY

Start of Block: Default Question Block

Pediatric Dentists' Management of Permanent Teeth Needing Endodontic Treatment

Thank you for your interest in this project! The purpose of this survey is to evaluate practice patterns among pediatric dentists regarding endodontic management of permanent teeth in pediatric dental patients.

Page Break

Pediatric Dentists' Management of Permanent Teeth Needing Endodontic Treatment

The purpose of this research study is to evaluate practice patterns among pediatric dentists regarding endodontic management of permanent teeth in pediatric dental patients.. You are being asked to take part in a research study because you are a pediatric dentist or currently completing pediatric dentistry residency.

Being in a research study is completely voluntary. You can choose not to be in this research study. You can also say yes now and change your mind later.

If you agree to take part in this research, you will be asked to answer questions regarding your clinical practice patterns for managing pediatric patients needing endodontic treatment on permanent teeth; answer basic demographic questions about you and your training. Your participation in this study will take about 10 minutes. We expect that all pediatric dentist members of the American Academy of Pediatric Dentistry (9,800 people) will take part in this research study.

You can choose not to answer any question you do not wish to answer. You can also choose to stop taking the survey at any time. You must be at least 18 years old to participate. If you are younger than 18 years old, please stop now.

The possible risks to you in taking part in this research are potential loss of confidentiality of data.

To protect your identity as a research subject, no identifiable information will be collected.

If you have any questions about this research, please contact the Investigator named at the top of this form by calling 919-537-3200 or emailing caroline_sawicki@unc.edu. If you have questions or concerns about your rights as a research subject, you may contact the UNC Institutional Review Board at 919-966-3113 or by email to IRB_subjects@unc.edu.

By proceeding with the survey, you are consenting to participate in the study.

Page Break

UNC SCHOOL OF DENTISTRY

First, please answer a question to ensure that you're eligible.

Page Break

UNC SCHOOL OF DENTISTRY

Are you currently completing or have already completed specialized training in pediatric dentistry from a CODA-approved program in the United States?

- ○ Yes
- ○ No

Skip To: End of Survey If = 2

Page Break

Great- you're eligible to complete the survey!

Page Break

These first questions ask about you and your training.

Page Break

Q1 How many years have you been practicing pediatric dentistry?

- ◯ Currently in pediatric dentistry residency
- ◯ 0-2 years post residency
- ◯ 2-5 years post residency
- ◯ 5-10 years post residency
- ◯ 10+ years post residency

Q2 How many days per week do you practice or encounter pediatric dentistry?

- ◯ 0
- ◯ 1
- ◯ 2
- ◯ 3
- ◯ 4
- ◯ 5
- ◯ 6+

UNC SCHOOL OF DENTISTRY

Q3 What type of clinical setting do you primarily work in?

- ○ Community clinic or federally qualified health center
- ○ Private practice
- ○ Hospital
- ○ Academic institution
- ○ Other; please describe ____

Q4 Which region do you primarily practice in?

- ○ Northeast
- ○ Midwest
- ○ West
- ○ Southeast
- ○ Southwest

Q5 In addition to pediatric dentistry, what other dental training have you received? *Check all that apply.*

- ☐ General Practice Residency
- ☐ Advanced Education in General Dentistry
- ☐ Oral Medicine
- ☐ Orofacial Pain
- ☐ Dental Anesthesiology
- ☐ Oral and Maxillofacial Surgery
- ☐ Oral and Maxillofacial Pathology
- ☐ Orthodontics
- ☐ Endodontics
- ☐ Prosthodontics
- ☐ Periodontics
- ☐ Dental Public Health
- ☐ Other; please describe

____

- ☐ None

Q6 Which range does your age fall into?

- ◯ Between 25 and 35 years
- ◯ Between 36 and 45 years
- ◯ Between 46 and 55 years
- ◯ Between 56 and 65 years
- ◯ Over 66 years
- ◯ Prefer not to answer

Q7 Which gender do you identify with?

- ◯ Male
- ◯ Female
- ◯ Non-binary / third gender
- ◯ Prefer not to answer

Q8 Which ethnicity do you identify with?

- ◯ Hispanic or Latinx
- ◯ Not Hispanic or Latinx
- ◯ Unsure
- ◯ Prefer not to answer

Q9 What race do you identify with?

- ○ White
- ○ Black or African American
- ○ American Indian or Alaska Native
- ○ Asian
- ○ Native Hawaiian or Other Pacific Islander
- ○ Other
- ○ Unsure
- ○ Prefer not to answer

Page Break

For the next set of questions, please describe your current clinical encounters.

Page Break

UNC
SCHOOL OF DENTISTRY

Q10 Do you perform any endodontic treatments (direct pulp cap, root canal therapy, apexification, etc.) for permanent teeth in pediatric patients in your practice?

- ○ Yes
- ○ No

Display This Question:

If Q10 = 54

Q11 What type of endodontic treatments on permanent teeth do you perform in your practice? *Check all that apply.*

- ☐ Direct pulp cap
- ☐ Partial or full pulpotomy
- ☐ Root canal therapy
- ☐ Apexogenesis or apexification
- ☐ Revascularization or pulpal regeneration
- ☐ Other; please describe ________________

Display This Question:

If Q10 = 54

Q11 Of the endodontic treatments you perform in practice, which teeth do you treat? *Check all that apply.*

- ▢ Anterior teeth
- ▢ Premolars
- ▢ Molars

Display This Question:

If Q10 = 55

Q14 For your pediatric patients requiring endodontic treatment on permanent teeth, who do you usually refer to?

- ○ Endodontist
- ○ General dentist
- ○ Oral surgeon
- ○ Another pediatric dentist
- ○ Other; please describe ____

Q15 On average, what percentage of pediatric patients requiring endodontic treatment on permanent teeth do you refer to an outside provider?

- ○ 0-25%
- ○ 26-50%
- ○ 51-75%
- ○ 76-100%

Q16 What are the main reasons for referring pediatric endodontic cases involving permanent dentition out of your practice? *Check all that apply.*

- ☐ Complexity of the case
- ☐ Limited resources
- ☐ Patient management concerns
- ☐ Patient insurance coverage
- ☐ Minimal exposure/experience in endodontics during pediatric dentistry residency
- ☐ Other; please describe ____

Page Break

The next set of questions asks about trauma cases in pediatric patients that involve the permanent dentition.

Page Break

Q17 How often do you encounter pediatric patients with traumatic dental injuries to permanent teeth in your practice?

- ○ Less than once a week
- ○ 1-2 times per week
- ○ 3-4 times per week
- ○ 5+ times per week

Q18 What percentage of trauma cases that you encounter involve the need for endodontic treatment in permanent teeth?

- ○ 0-10%
- ○ 11-25%
- ○ 26-50%
- ○ 51-75%
- ○ 76-100%

Q19 In your experience, what age group most commonly presents with traumatic injuries in permanent teeth requiring endodontic treatment?

- ○ 6-8 years
- ○ 9-12 years
- ○ 13-16 years
- ○ 17 years or older

UNC SCHOOL OF DENTISTRY

Q20 When a patient presents with a trauma in the permanent dentition involving need for endodontic treatment, do you perform the initial consult and evaluation or do you immediately refer them to a specialist?

- ◯ I perform the initial consult and evaluation
- ◯ I sometimes perform the initial consult and evaluation, depending on the complexity of the case
- ◯ I immediately refer out to a specialist without an initial consult
- ◯ Other; please describe ____

Page Break

These next questions pertain to educational resources for the management of permanent teeth needing endodontic treatment.

Q21 Which of these educational resources would be most helpful for you as a pediatric dentist to best manage pediatric endodontic cases involving permanent dentition? *Check all that apply.*

▢ More studies to determine the prevalence of permanent teeth needing endodontic treatment in pediatric patients

▢ Continuing education courses and training focused on permanent teeth needing endodontic treatment in pediatric patients

▢ More lectures at the AAPD Annual Session dedicated to endodontic management of permanent teeth

▢ Annual joint symposia/meeting of AAPD and American Association of Endodontics (AAE)

▢ Other; please describe ____

Q22 Besides pediatric dentistry residency, what additional training have you received that is relevant to managing permanent teeth that need endodontic treatment in pediatric patients? *Check all that apply.*

- ☐ Additional postgraduate specialty training
- ☐ Continuing education courses
- ☐ Simulation training
- ☐ Attendance at national and/or international conferences
- ☐ Other; please describe ____

Page Break

For the last question, please state your level of agreement with the following statements.

Page Break

Q23

Please state your agreement level (0=strongly disagree, 100=strongly agree).

	0	10	20	30	40	50	60	70	80	90	100
I received sufficient training during my pediatric dentistry residency on performing endodontic treatment for permanent teeth in pediatric patients											
I feel comfortable performing endodontic treatment on permanent teeth in pediatric patients											
I feel comfortable managing traumatic dental injuries involving permanent teeth in pediatric patients											
I would be interested in learning more about how to treat pediatric endodontic cases involving permanent dentition											

Page Break

Please share any other thoughts or feedback related to the management of pediatric endodontic cases involving permanent dentition.

____

Page Break

End of Block: Default Question Block
